# Supplementary material for: Deep-learning-enabled brain hemodynamic mapping using resting-state fMRI
Source: NPJ Digit Med. 2023 Jun 21;6:116. doi: 10.1038/s41746-023-00859-y (PMC10284915; doi:10.1038/s41746-023-00859-y)
Supplement: Supplementary file 1 — Supplementary Information [file 41746_2023_859_MOESM1_ESM.pdf]

Supplementary Information

Deep-learning-enabled Brain Hemodynamic Mapping Using Resting-state fMRI

Xirui Hou[1,2], Pengfei Guo[3], Puyang Wang[4], Peiying Liu[5], Doris D.M. Lin[2], Hongli Fan[1,2], Yang Li[2], Zhiliang Wei[2,6], Zixuan Lin[2], Dengrong Jiang[2], Jin Jin[7], Catherine Kelly[8], Jay J. Pillai[2,9], Judy Huang[9], Marco C. Pinho[10], Binu P. Thomas[10], Babu G. Welch[11,12], Denise C. Park[12], Vishal M. Patel[3,4], Argye E. Hillis[8], and Hanzhang Lu[1,2,6*]

[1]Department of Biomedical Engineering, Johns Hopkins University School of Medicine, Baltimore, MD, USA

[2]The Russell H. Morgan Department of Radiology and Radiological Science, Johns Hopkins University School of Medicine, Baltimore, MD, USA

[3]Department of Computer Science, Johns Hopkins University, Baltimore, MD, USA

[4]Department of Electrical and Computer Engineering, Johns Hopkins University, Baltimore, MD, USA

[5]Department of Diagnostic Radiology and Nuclear Medicine, University of Maryland School of Medicine, Baltimore, MD, USA

[6]F.M. Kirby Research Center for Functional Brain Imaging, Kennedy Krieger Institute, Baltimore, MD, USA

[7]Department of Biostatistics, Johns Hopkins Bloomberg School of Public Health, Baltimore, MD, USA

[8]Department of Neurology, Johns Hopkins University School of Medicine, Baltimore, MD, USA

[9]Department of Neurosurgery, Johns Hopkins University School of Medicine, Baltimore, MD, USA

[10]Department of Radiology, UT Southwestern Medical Center, Dallas, TX, USA

[11]Department of Neurologic Surgery, UT Southwestern Medical Center, Dallas, TX, USA

[12]Center for Vital Longevity, School of Behavioral and Brain Sciences, University of Texas at Dallas, Dallas, TX, USA

***Correspondence:** Hanzhang Lu, Ph.D. The Russell H. Morgan Department of Radiology and Radiological Science, Johns Hopkins School of Medicine, 600 N. Wolfe Street, Baltimore, MD, USA, 21287, Email: hanzhang.lu@jhu.edu

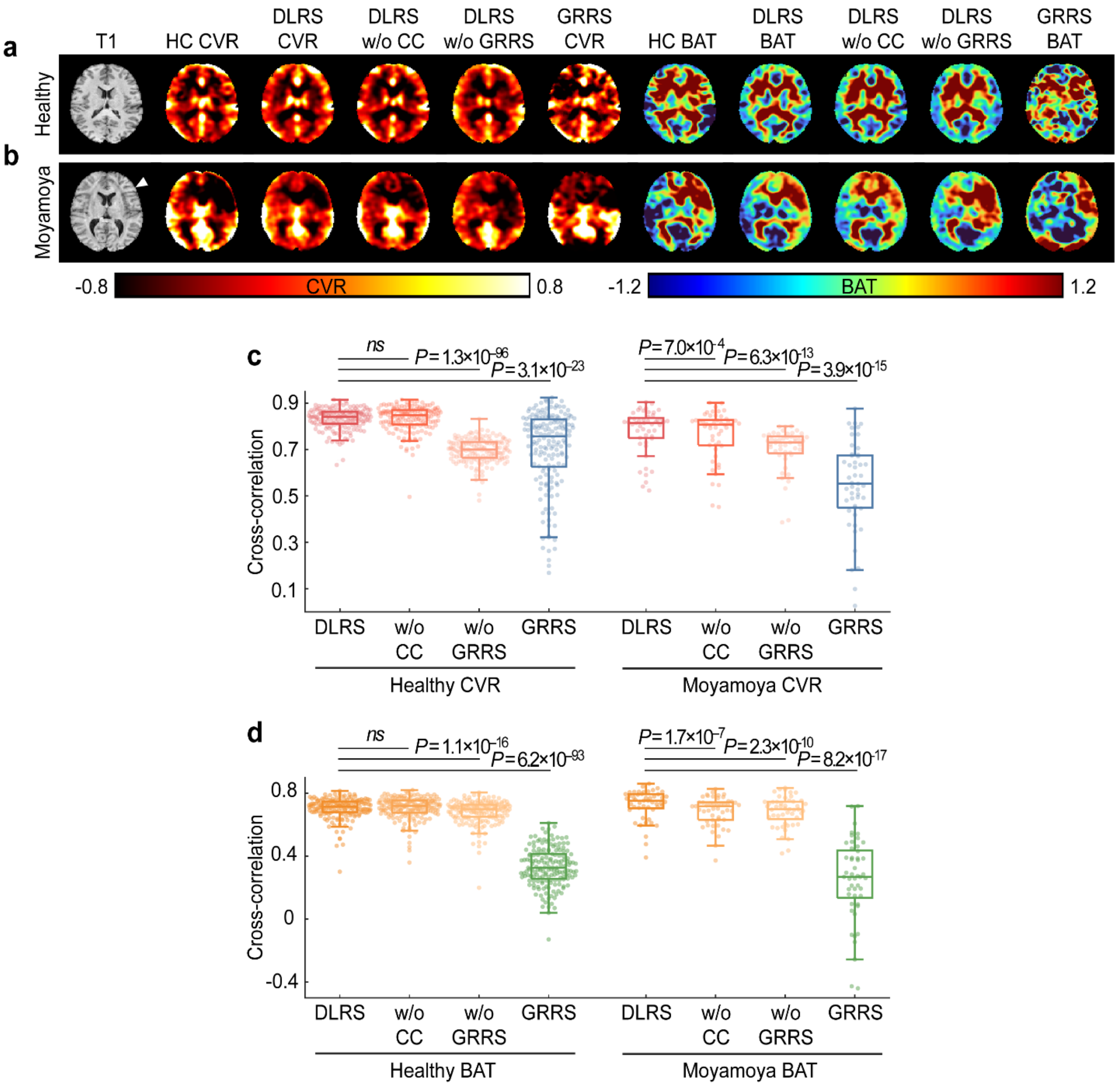

Supplementary Figure 1 | Examples and quantitative metrics of the ablation study. a, b, Representative cerebrovascular reactivity (CVR) and bolus arrival time (BAT) images of a healthy volunteer **(a)**, and a Moyamoya disease patient **(b).** From left to right, the images are T1-weighted images, hypercapnic (HC) CVR, deep-learning resting-state (DLRS) CVR, DLRS CVR without cross-correlation inputs, DLRS CVR without global-regression inputs, global-regression resting-state (GRRS) CVR, HC BAT, DLRS BAT, DLRS BAT without cross-correlation inputs, DLRS BAT without GRRS inputs, and GRRS BAT. **c,** Boxplots denote Pearson cross-correlation between RS CVR images and HC CVR images in healthy and Moyamoya disease participants. The ablated parametric maps revealed a lower correlation with the reference HC maps. The line, box, and whiskers in the boxplots represent the median, the interquartile range (IQR), and 1.5 times the IQR, respectively. **d,** Boxplots denote Pearson cross-correlation between RS BAT images and HC BAT images.

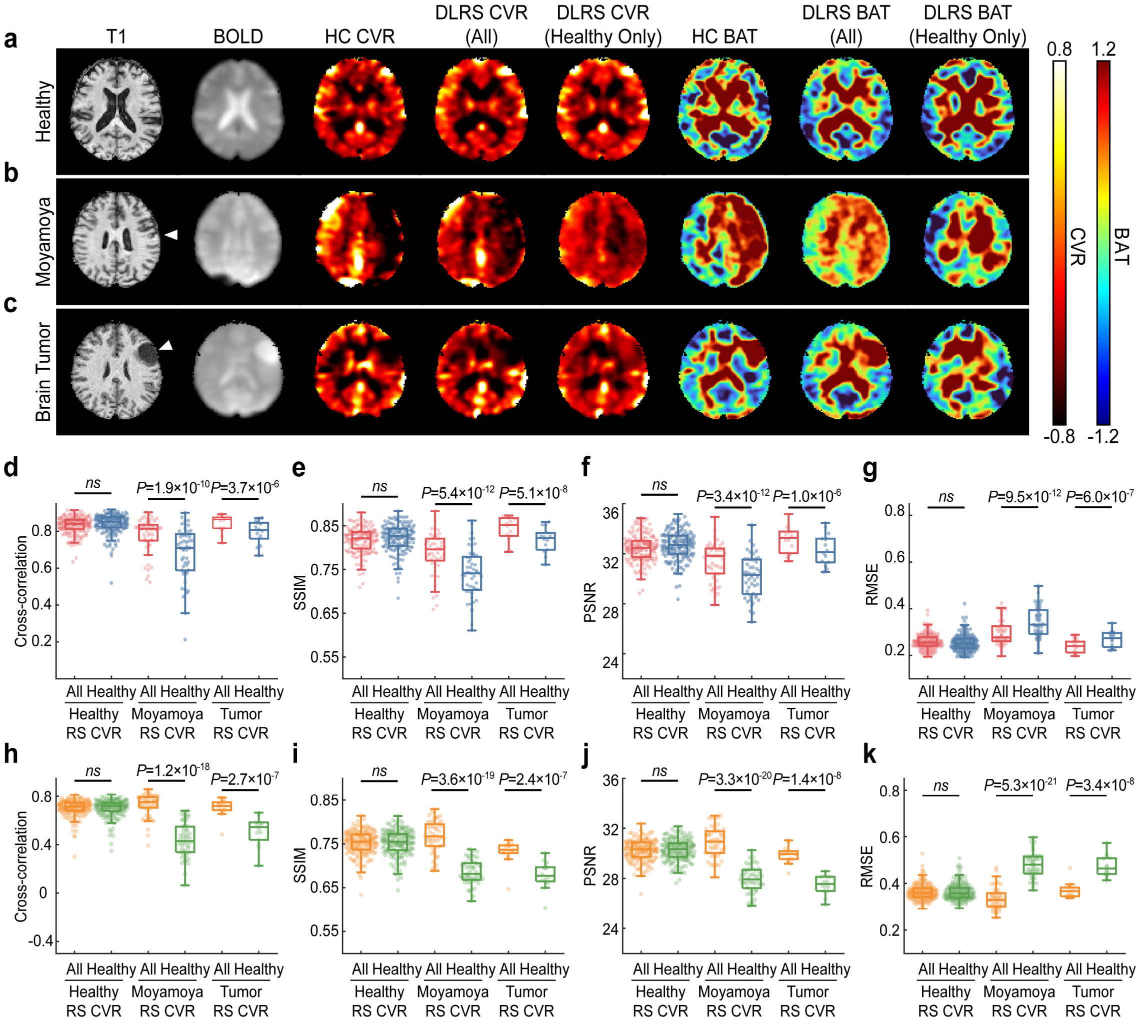

Supplementary Figure 2 | The performance of deep-learning network trained exclusively on healthy group. a-c, Representative cerebrovascular reactivity (CVR) and bolus arrival time (BAT) images of a healthy volunteer (a), Moyamoya disease patient (b), and brain tumor patient (c). From left to right, the images are T1-weighted images, raw BOLD images, hypercapnic (HC) CVR and BAT images, as well as deep-learning resting-state (DLRS) CVR and BAT images trained on all groups and healthy group. **d-g,** The boxplots display the similarity between deep-learning resting-state CVR maps and ground-truth HC CVR maps. Two types of resting-state CVR maps are studied: those trained on all groups and those specifically trained on the healthy group. Four similarity indices are studied, including Pearson cross-correlation **(d)**, structure similarity index metric (SSIM) **(e)**, peak signal-to-noise ratio (PSNR) **(f)**, root-mean-square error (RMSE) **(g)**. The line within the boxplots represents the median, the box represents the interquartile range (IQR), and the whiskers are 1.5 times the IQR. **h-k**, the boxplots display the similarity between deep-learning resting-state BAT maps and ground-truth HC BAT maps.

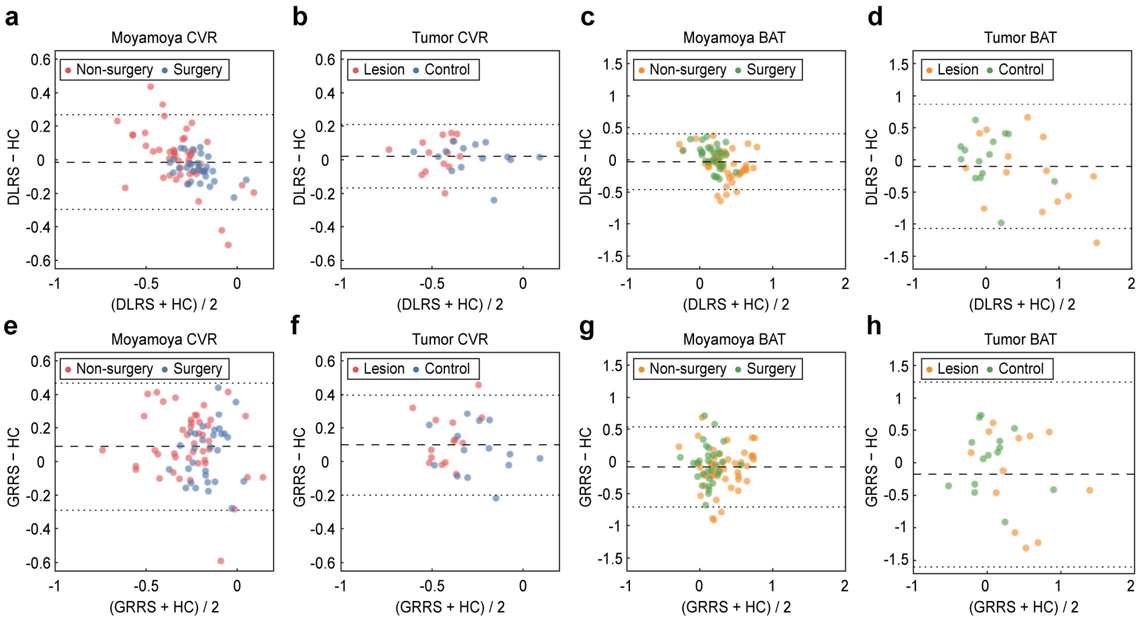

Supplementary Figure 3 | Bland-Altman plots comparing the results of deep-learning resting-state (DLRS) and global-regression resting-state (GRRS) approaches to hypercapnic (HC) results. Top row: DLRS results. Bottom row: GRRS results. Left half: CVR results. Right half: BAT results.

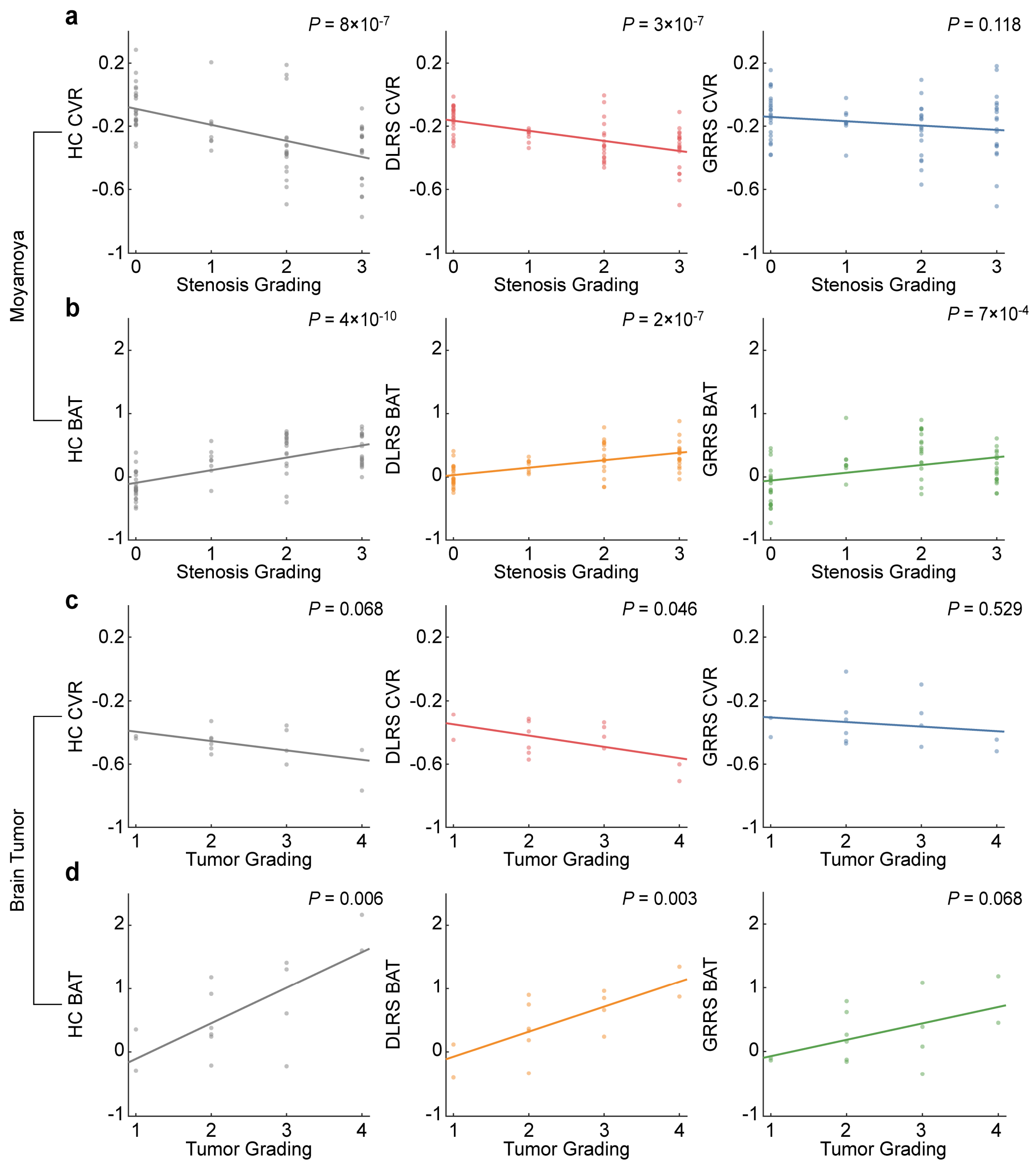

Supplementary Figure 4 | Associations between cerebrovascular physiological maps and clinical variables. a, Scatter plots between regional CVR and arterial stenosis grade in Moyamoya patients, as rated in the middle cerebral artery (MCA). Plots are shown for hypercapnic cerebrovascular reactivity (HC CVR, left), deep-learning resting-state (DLRS CVR, middle) and global-regression resting-state (GRRS CVR, right). More severe stenosis is associated with lower CVR. **b,** Scatter plot between BAT and arterial stenosis grade. More severe stenosis is associated with longer BAT. **c,** Scatter plots between regional CVR and WHO tumor grade. **d,** Scatter plots between regional BAT and WHO tumor grade.

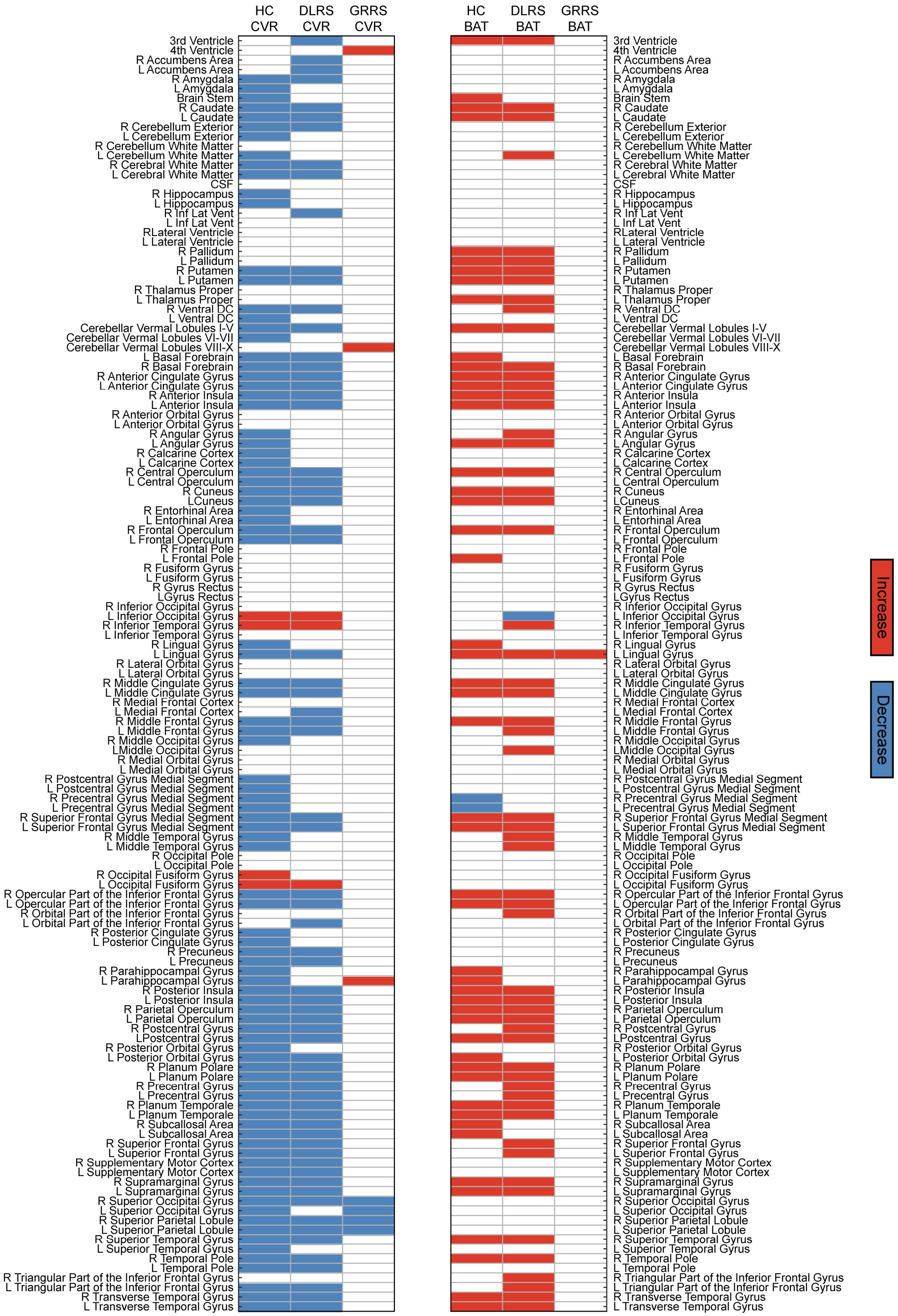

Supplementary Figure 5 | Age-related differences in deep-learning resting-state cerebrovascular reactivity (DLRS CVR) and bolus arrival time (DLRS BAT) across the lifespan. From left to right, the heat maps represent the age differences associated with

hypercapnic (HC) CVR, DLRS CVR, global-regression resting-state (GRRS) CVR, HC BAT, DLRS BAT and GRRS BAT. Cell color denotes model type: linear increasing (Red) and linear decreasing (Blue). All models are added gender as covariate and controlled the false discovery rate (FDR, $q<0.05$).

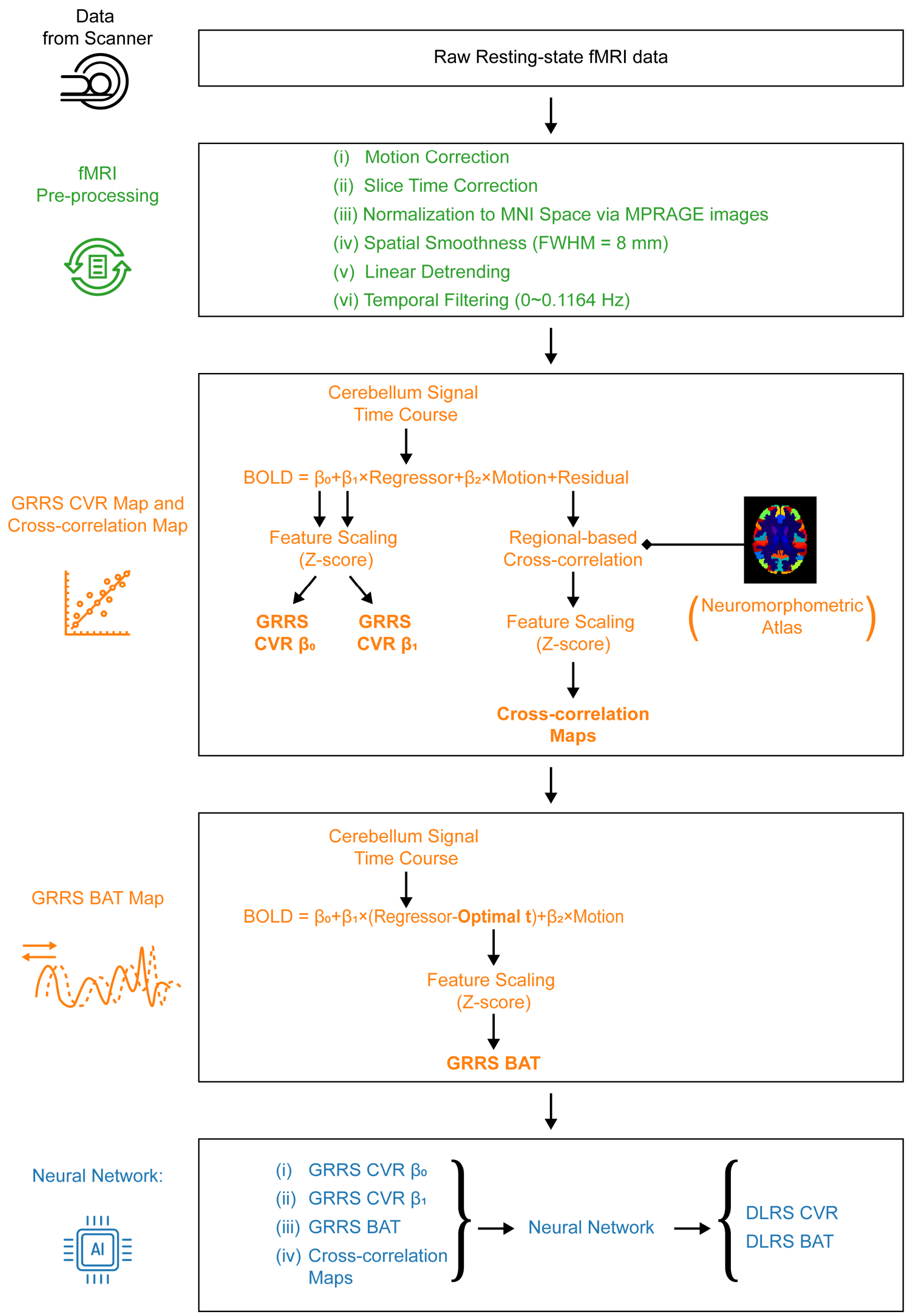

Supplementary Figure 6 | Illustration of the analysis pipelines of deep-learning resting-state cerebrovascular reactivity (DLRS CVR) and bolus arrival time (DLRS BAT) using resting-state fMRI data.

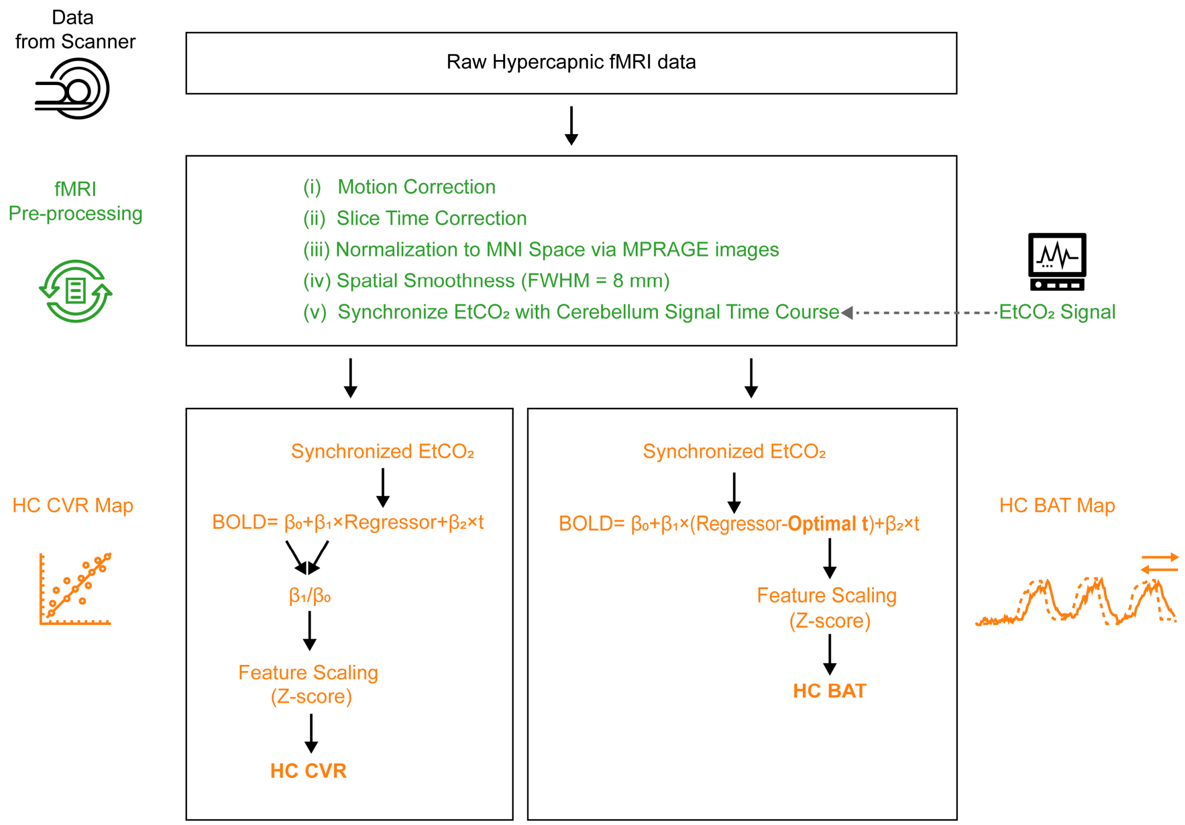

Supplementary Figure 7 | Illustration of the analysis pipelines of hypercapnic cerebrovascular reactivity (HC CVR) and bolus arrival time (HC BAT).

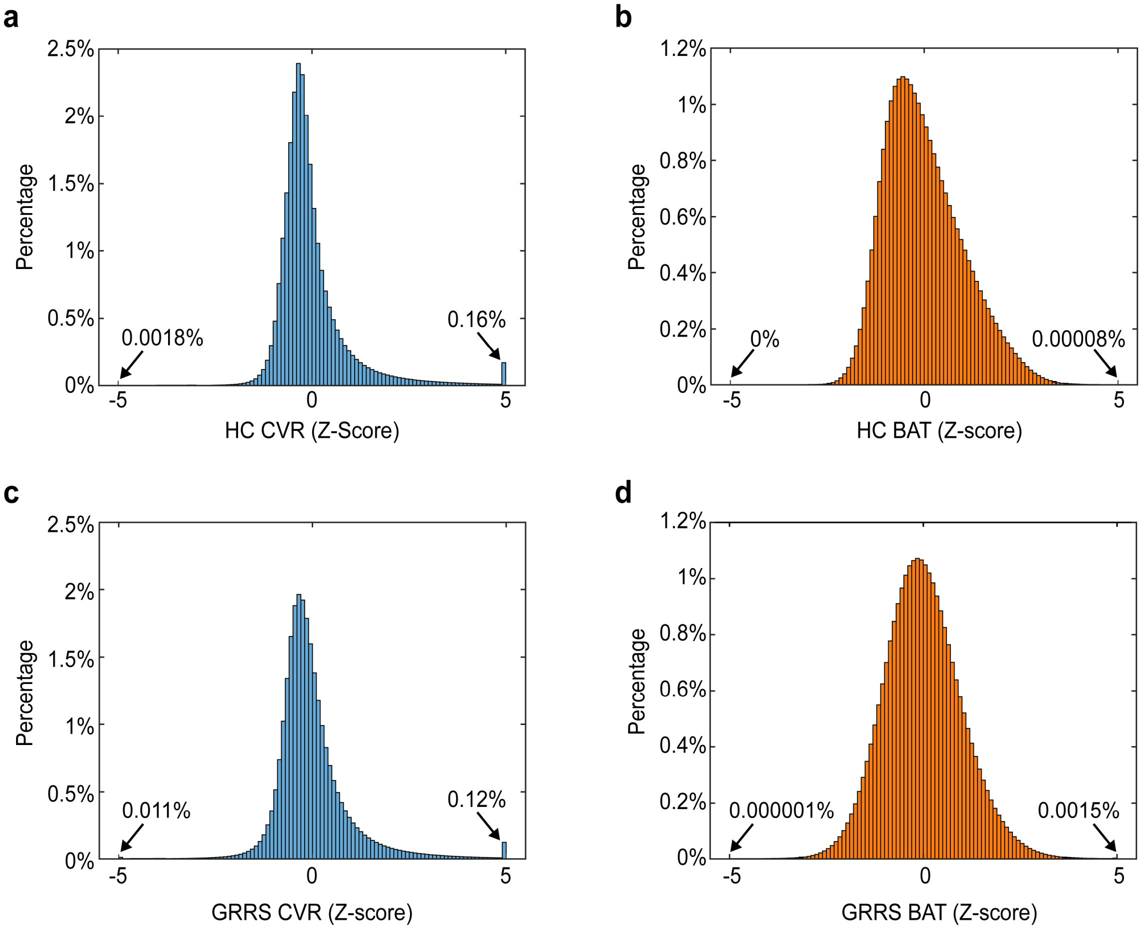

Supplementary Figure 8 | Histograms of cerebrovascular reactivity (CVR) and bolus arrival time (BAT) after imaging clipping to a range of less than ±5. a, Clipped distribution of hypercapnic (HC) CVR voxel-wise values after conversion to z-score. **b,** Clipped distribution of HC BAT voxel-wise values after conversion to z-score. **c,** Clipped distribution of global-regression resting-state (GRRS) CVR. **d,** Clipped distribution of GRRS BAT.

Supplementary Table 1 | Demographic and clinical data of Moyamoya patients.

Subject number	Age (years)	Sex	MRA stenosis grade for cerebral arteries							
			R ICA	L ICA	R MCA	L MCA	R ACA	L ACA	R PCA	L PCA
1	18	F	0	3	0	2	0	0	0	0
2	21	F	1	0	1	0	0	0	0	0
3	24	F	3	0	2	0	0	2	0	0
4	24	F	3	1	3	3	0	0	0	0
5	24	F	0	3	0	2	0	0	0	0
6	25	F	0	1	0	2	0	0	0	0
7	30	F	3	1	3	3	1	0	0	0
8	30	F	1	3	2	3	1	2	0	0
9	30	F	2	2	3	2	1	0	0	0
10	31	M	3	2	3	3	2	1	0	0
11	32	F	0	0	0	2	0	0	0	0
12	32	M	2	2	2	2	2	2	0	0
13	34	F	2	1	3	1	2	1	1	1
14	34	F	3	3	3	3	1	2	0	0
15	35	M	2	2	0	2	1	1	0	0
16	35	F	0	3	0	3	0	0	0	0
17	36	F	0	0	0	2	0	0	0	0
18	37	F	0	0	0	2	0	0	0	0
19	38	F	2	0	2	0	1	0	0	0
20	38	M	1	1	3	2	0	1	0	0
21	38	F	1	2	3	1	0	0	1	0
22	38	F	0	2	0	3	0	1	0	0
23	38	F	2	1	1	2	0	0	0	0
24	39	F	2	3	2	3	2	1	1	2
25	39	F	2	3	2	3	2	1	1	2
26	40	F	2	3	2	3	2	1	1	2
27	41	F	2	2	2	2	0	0	1	0
28	41	F	2	2	0	2	0	2	0	0
29	42	F	3	3	3	3	2	2	0	0
30	45	F	2	0	1	0	0	0	0	0
31	45	F	1	0	1	0	0	0	0	0
32	46	M	3	3	3	0	0	0	1	0
33	46	F	0	0	0	3	0	0	0	0
34	48	F	0	3	0	2	2	1	0	0
35	49	F	1	3	2	3	0	1	0	0
36	49	F	2	2	2	3	0	2	0	0
37	50	F	1	0	0	0	0	0	0	0
38	50	F	2	1	1	3	0	2	0	0
39	50	F	2	1	1	3	1	2	0	0
40	51	F	3	3	2	3	1	2	0	0
41	51	F	2	2	3	3	1	2	0	0
42	52	F	3	3	3	3	2	0	0	0
43	52	F	1	1	3	2	0	2	0	0
44	53	M	0	2	0	3	2	0	0	0
45	60	F	2	1	1	2	0	0	0	0
46	60	F	2	1	1	3	0	1	0	0
47*	62	F	-	-	-	-	-	-	0	0
48	64	F	1	1	0	1	2	1	0	0
49	72	F	1	1	0	3	2	0	1	0

*In subject 47, the MR angiograph was postioned too high to grade the stenosis of some cerebral arteries.

Supplementary Table 1 | Demographic and clinical data of Moyamoya patients.

Supplementary Table 2 | Demographic and clinical data of brain tumor patients.				
Subject number	Age (years)	Sex	WHO Grade	Histology
1	21	M	3	Anaplastic astrocytoma
2	24	M	2	Astrocytoma
3	25	F	2	Oligodendroglioma
4	26	F	3	Astrocytoma
5	33	M	4	Glioblastoma
6	34	M	3	Anaplastic glioma
7	35	M	2	Diffuse astrocytoma
8	41	F	2	Diffuse astroctoma
9	42	M	1	Glioma
10	46	M	1	Glioma
11	58	M	4	Glioblastoma
12	59	F	2	Infiltrating glioma
13	68	M	2	Infiltrating glioma
14	81	F	3	Astrocytoma

Supplementary Table 2 | Demographic and clinical data of brain tumor patients.

**Supplementary Table 3 |** Demographic and clinical data of stroke patients used for clinical test.				
Subject number	Age (years)	Sex	Time of scan post-stroke (Weeks)	NIHSS
1	27	F	0	3
2	28	F	51	4
3	33	F	22	11
4	36	M	9	6
5	39	F	31	
6	39	F	1	2
7	40	M	49	1
8	46	M	2	1
9	46	F	32	9
10	47	M	1	0
11	49	M	52	0
12	50	M	1	1
13	50	M	12	1
14	52	M	0	1
15	54	F	0	11
16	54	F	20	3
17	54	M	59	2
18	56	M	56	2
19	56	M	24	2
20	57	F	24	1
21	57	M	10	2
22	57	F	0	2
23	59	M	2	1
24	60	F	4	5
25	60	M	52	4
26	60	M	30	23
27	61	F	63	3
28	61	M	64	5
29	62	M	16	6
30*	63	F	12	-
31	63	M	7	0
32	64	M	0	2
33	65	M	0	1
34	66	M	0	2
35	71	F	11	2
36	74	M	17	5
37	76	M	0	2
38	87	F	0	10

* In subject 30, the subject was refered for clinical research sub-acutely and not seen by neurologist.

Supplementary Table 3 | Demographic and clinical data of stroke patients used for clinical test.

Supplementary Table 4 | Demographic and clinical data of stroke patients used for reproducibility test.				
Subject number	Age (years)	Sex	Time of scan post-stroke (Weeks)	NIHSS
1	24	F	8	0
2	34	M	0	0
3	36	M	16	20
4	37	M	1	3
5	41	F	55	4
6	42	F	16	2
7	46	M	62	0
8	48	M	30	3
9	52	M	30	7
10	53	M	14	1
11	56	M	0	1
12	56	M	34	2
13	56	M	42	5
14	57	M	51	1
15	57	M	31	7
16	57	M	28	11
17	60	F	0	9
18	63	F	33	1
19	63	F	0	1
20	64	M	12	0
21	65	M	14	5
22	66	M	1	0
23	66	F	62	6
24	68	M	21	1
25	70	F	11	7
26	70	F	0	4
27	70	M	19	4
28	72	M	1	1
29	76	F	20	8
30	80	F	1	1

Supplementary Table 4 | Demographic and clinical data of stroke patients used for reproducibility test.

Table S5. Architectural details of the deep-learning network used in this work.

Module	Block	Layer	Input Layer	Input Size	Num of Filters	Kernal Size	Stride	Padding
Primary Encoder	P_Conv1	Conv+ReLU+BN	Images	3x96x112	64	3	1	1
		Conv+ReLU+BN	Prev	64x96x112	64	3	1	1
		MaxPool	Prev	64x96x112	1	2	2	0
	P_Conv2	Conv+ReLU+BN	P_Conv1	64x48x56	128	3	1	1
		Conv+ReLU+BN	Prev	128x48x56	128	3	1	1
		MaxPool	Prev	128x48x56	1	2	2	0
	P_Conv3	Conv+ReLU+BN	P_Conv2	128x24x28	256	3	1	1
		Conv+ReLU+BN	Prev	256x24x28	256	3	1	1
		MaxPool	Prev	256x24x28	1	2	2	0
	P_Conv4	Conv+ReLU+BN	P_Conv3	256x12x14	512	3	1	1
		Conv+ReLU+BN	Prev	512x12x14	512	3	1	1
		MaxPool	Prev	512x12x14	1	2	2	0
	P_Conv5	Conv+ReLU+BN	P_Conv4	512x6x7	1024	3	1	1
		Conv+ReLU+BN	Prev	1024x6x7	1024	3	1	1
Supplementary Encoder	S_Conv1	Conv+ReLU+BN	Images	133x96x112	64	3	1	1
		Conv+ReLU+BN	Prev	64x96x112	64	3	1	1
		MaxPool	Prev	64x96x112	1	2	2	0
	S_Conv2	Conv+ReLU+BN	S_Conv1	64x48x56	128	3	1	1
		Conv+ReLU+BN	Prev	128x48x56	128	3	1	1
		MaxPool	Prev	128x48x56	1	2	2	0
	S_Conv3	Conv+ReLU+BN	S_Conv2	128x24x28	256	3	1	1
		Conv+ReLU+BN	Prev	256x24x28	256	3	1	1
		MaxPool	Prev	256x24x28	1	2	2	0
	S_Conv4	Conv+ReLU+BN	S_Conv3	256x12x14	512	3	1	1
		Conv+ReLU+BN	Prev	512x12x14	512	3	1	1
		MaxPool	Prev	512x12x14	1	2	2	0
	S_Conv5	Conv+ReLU+BN	S_Conv4	512x6x7	1024	3	1	1
		Conv+ReLU+BN	Prev	1024x6x7	1024	3	1	1

Module	Block	Layer	Input Layer	Input Size	Num of Filters	Kernal Size	Stride	Padding
CVR Decoder	C_DeConv1	Transpose Conv	P_Conv5+S_Conv5	2048x6x7	512	2	2	0
		Concatenate	P_Conv4+S_Conv4+Prev	1536x12x14	-	-	-	-
		Conv+ReLU+BN	Prev	1536x12x14	512	3	1	1
		Conv+ReLU+BN	Prev	512x12x14	512	3	1	1
	C_DeConv2	Transpose Conv	C_DeConv1	512x12x14	256	2	2	0
		Concatenate	P_Conv3+S_Conv3+Prev	768x24x28	-	-	-	-
		Conv+ReLU+BN	Prev	768x24x28	256	3	1	1
		Conv+ReLU+BN	Prev	256x24x28	256	3	1	1
	C_DeConv3	Transpose Conv	C_DeConv2	256x24x28	128	2	2	0
		Concatenate	P_Conv2+S_Conv2+Prev	384x48x56	-	-	-	-
		Conv+ReLU+BN	Prev	384x48x56	128	3	1	1
		Conv+ReLU+BN	Prev	128x48x56	128	3	1	1
	C_DeConv4	Transpose Conv	C_DeConv3	128x48x56	64	2	2	0
		Concatenate	P_Conv1+S_Conv1+Prev	192x96x112	-	-	-	-
		Conv+ReLU+BN	Prev	192x96x112	64	3	1	1
		Conv+ReLU+BN	Prev	64x96x112	64	3	1	1
	C_Output	Conv+Tanh	C_DeConv4	64x96x112	1	1	1	0
BAT Decoder	B_DeConv1	Transpose Conv	P_Conv5+S_Conv5	2048x6x7	512	2	2	0
		Concatenate	P_Conv4+S_Conv4+Prev	1536x12x14	-	-	-	-
		Conv+ReLU+BN	Prev	1536x12x14	512	3	1	1
		Conv+ReLU+BN	Prev	512x12x14	512	3	1	1
	B_DeConv2	Transpose Conv	B_DeConv1	512x12x14	256	2	2	0
		+Concatenate	P_Conv3+S_Conv3+Prev	768x24x28	-	-	-	-
		Conv+ReLU+BN	Prev	768x24x28	256	3	1	1
		Conv+ReLU+BN	Prev	256x24x28	256	3	1	1
	B_DeConv3	Transpose Conv	B_DeConv2	256x24x28	128	2	2	0
		Concatenate	P_Conv2+S_Conv2+Prev	384x48x56	-	-	-	-
		Conv+ReLU+BN	Prev	384x48x56	128	3	1	1
		Conv+ReLU+BN	Prev	128x48x56	128	3	1	1
	B_DeConv4	Transpose Conv	B_DeConv3	128x48x56	64	2	2	0
		Concatenate	P_Conv1+S_Conv1+Prev	192x96x112	-	-	-	-
		Conv+ReLU+BN	Prev	192x96x112	64	3	1	1
		Conv+ReLU+BN	Prev	64x96x112	64	3	1	1
	B_Output	Conv+Tanh	B_DeConv4	64x96x112	1	1	1	0

Conv: Convolution, ReLU: Rectified linear unit, BN: Batch normalization, Tanh: Hyperbolic tangent, Prev: Previous layer output.

Supplementary Table 5 | Architectural details of the deep-learning network used in this work.
